# Medicinal plants and their derivatives for skin and hair: a Mediterranean perspective of women care

**DOI:** 10.1007/s00403-025-04202-1

**Published:** 2025-04-13

**Authors:** Latifa Bouissane, Yahya Elfardi, Sohaib Khatib, Ahmed Fatimi, Carla Pereira, Natália Cruz-Martins

**Affiliations:** 1 Molecular Chemistry, Materials and Catalysis Laboratory, Faculty of Sciences and Technologies, Sultan Moulay Slimane University, BP 523, 23000 Beni-Mellal, Morocco; 2 Chemical Science and Engineering Research Team (ERSIC), Department of Chemistry, Polydisciplinary Faculty of Beni Mellal (FPBM), Sultan Moulay Slimane University (USMS), Mghila Campus, P.O. Box 592, 23000 Beni Mellal, Morocco; 3 CIMO, La SusTEC, Instituto Politécnico de Bragança, Campus de Santa Apolónia, 5300-253 Bragança, Portugal; 4 Faculty of Medicine, University of Porto, 4200-319 Porto, Portugal; 5 Institute for Research and Innovation in Health (i3S), University of Porto, 4200-319 Porto, Portugal; 6 Life and Health Sciences Research Institute (ICVS), School of Medicine, University of Minho, Braga, Portugal

**Keywords:** Plant-based cosmetics, Mediterranean women care, Beautification, Prevention, Skin and hair diseases, Formulation

## Abstract

Since olden days, medicinal and aromatic plants have been consumed due to their well-known therapeutic, ornamental, culinary, and cosmetic properties. Plant-based cosmetics comprise a growing market offering brands of sustainable products with the ultimate goal of responding to the woman needs to boost their natural beauty, such as soothing, toning, moisturizing and protecting skin and hair. The increasing focus on body and beauty care, combined with a better understanding of the diverse biological effects of plants and their derivatives, has revitalized their significance in aesthetic, cosmetic, and dermatological contexts. Concurrently, the increasing prevalence of allergies and hypersensitivity reactions to synthetic additives commonly found in cosmetics and other skin products has spurred interest in seeking healthier and more efficacious natural alternatives. An intense investigation has been stated around the formulation of medicinal plants- and other plant-based cosmetic products for dermatological and aesthetic purposes. Similarly, a raising awareness by cosmetics’ manufacturers and related industries have been progressively stated, culminating with the emergence of a line of plant-based cosmetics increasingly safe and with reliable quality features for multiple purposes. Although a high demand for natural-based products for cosmetic purposes has been stated, further studies are required to deepening knowledge on their beneficial properties, safety and quality features and to identify the main limitations and likelihood of side effects occurrence. In this review, an outlook of the current scenario regarding the use of medicinal and aromatic plants as cosmetic ingredients in the formulation of skin care and other dermatological products traditionally used by the Mediterranean woman for prevention and even cure of skin and hair diseases or for beautification is provided.

**Graphical abstract:**

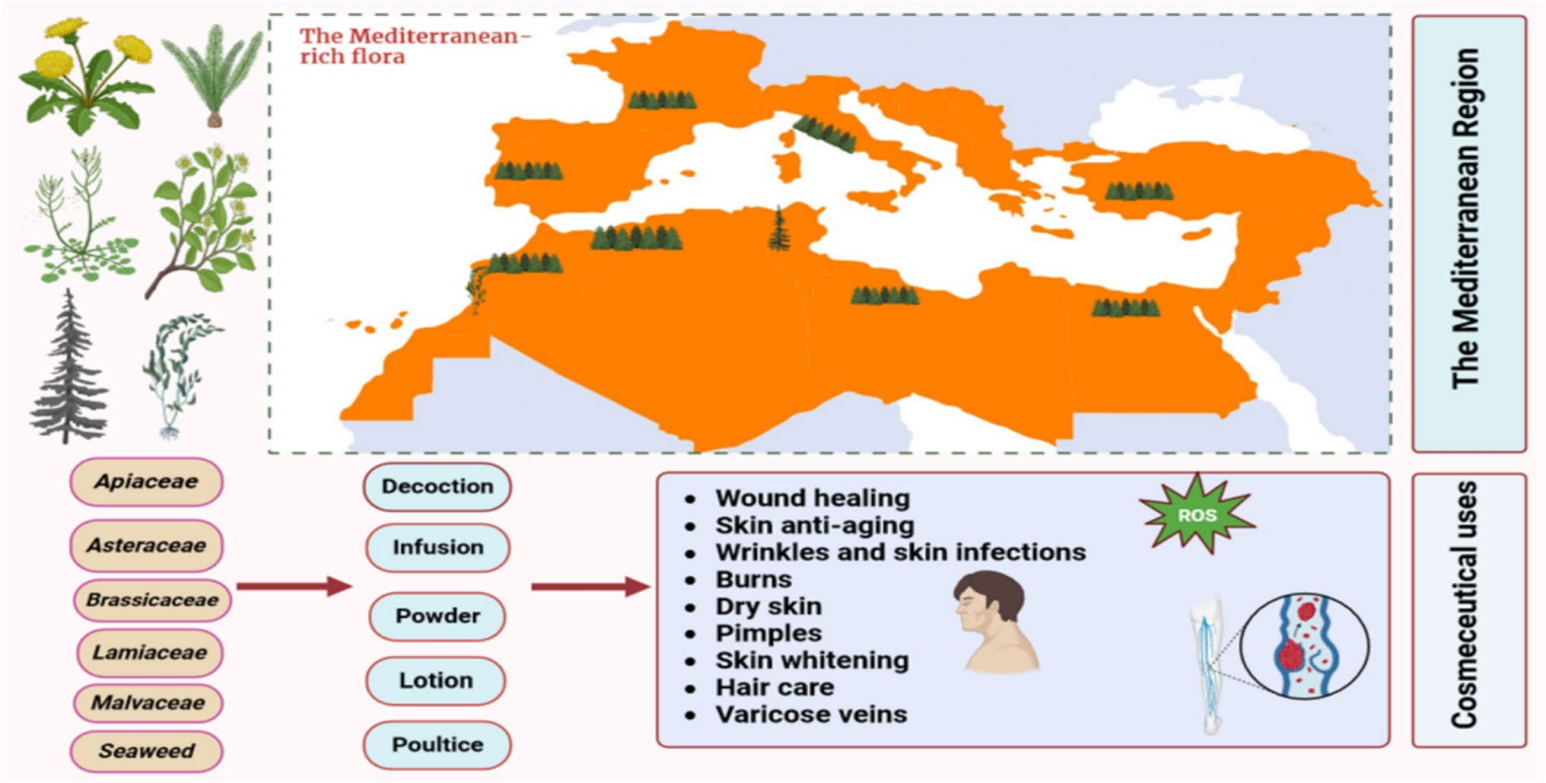

## Introduction

Medicinal plants have been widely used mainly to prevent and treat several maladies in the traditional medical practices. Thanks to its multipurpose, the strong demand of the medicinal plants has been propelled nationally and internationally [1–9]. Throughout the foregoing decades, Mediterranean women in different cultures and civilizations having lived in this area had led the spared of the use of aromatic and medicinal plants in their cosmetic preparations. Consequently, succeeding generations have inherited how to custom these plants in order to preserve their beauty. In fact, women in ancient Egyptian civilization had used famous eyes shadow called “Kohl”, also they used Henna to dye their hair [10]. To explain the massive use of medicinal plants in a 2% of the Earth´s land surface, botanists counted around 25,000 species, which represents 10% of the plants found on a planetary scale [11].

The definition of cosmetic products differs from one source to another although they are intended for beautification and personal care [12–14]. However, due to the growing demand from consumers, issues related to the importance of sustainability have become basic in the present time [15]. In fact, the world is facing the running down of the natural resources because of the population upward and to its strong demand for natural products [16, 17]. Therefore, there is a need to contribute effectively towards sustainability through finding a balance between improving the lives of people and preserving natural resources [18]. Nowadays, the consumers ask more for green living encouraging the cosmetic manufacturing companies to go forward to produce plant-based beauty products. So, with the development of science; scientists are doing research with the aim of answering the question: does the use of these plants really have a cosmetic effect? Based on the above, this review highlights the interest in using the natural products from the Mediterranean region and sheds light on the Mediterranean woman’s secret beauties.

## Methodology section

**Fig. 1:**
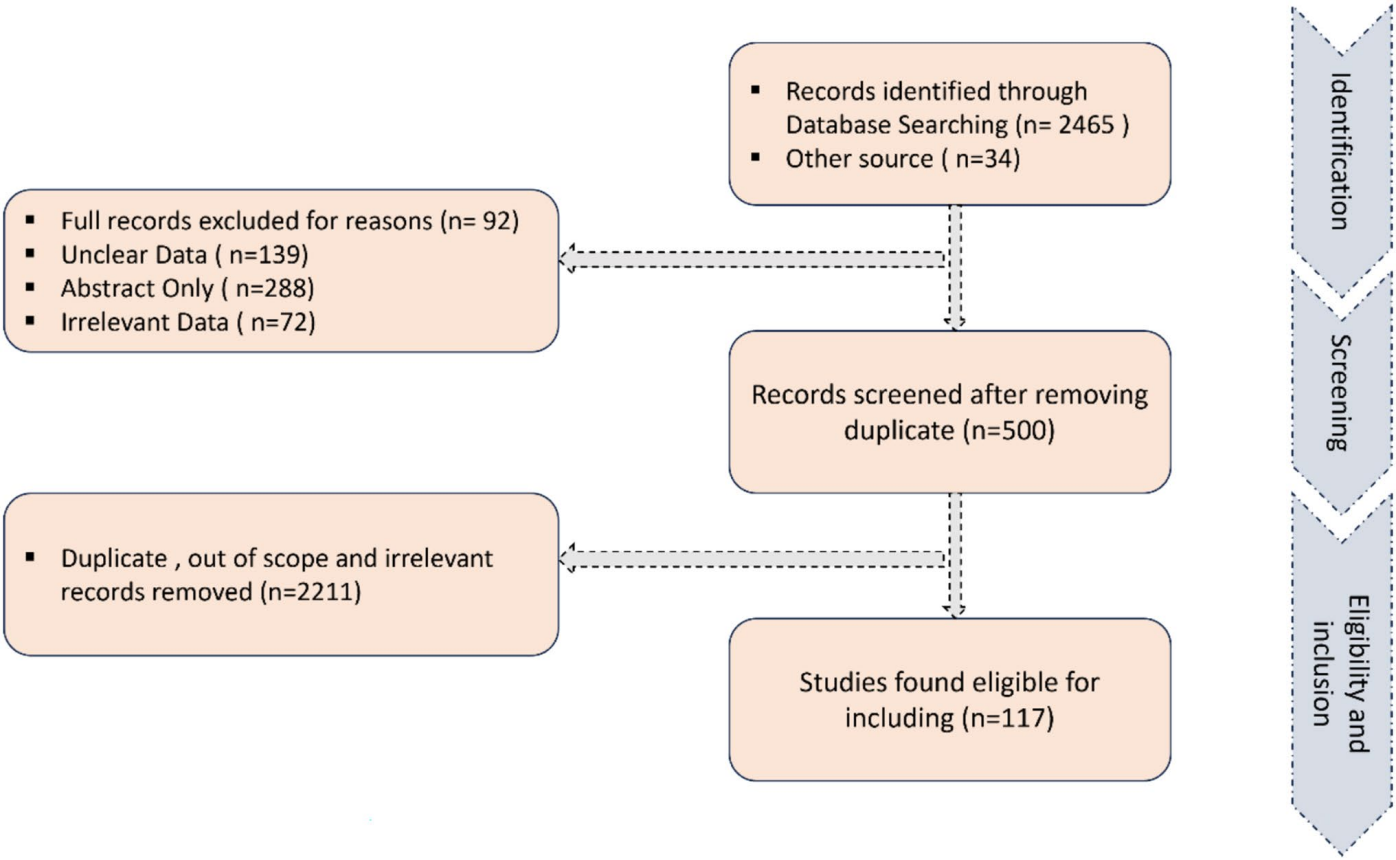
PRISMA flow diagram

All-inclusive literature search was led using various academic databases (e.g., PubMed, Scopus, Google Scholar, Science Direct, among others) to identify relevant studies published in peer-reviewed journals between 1988 and 2025. Keywords, such as "cosmeceutical plants," "medicinal plants," "skin care," "wound healing," "Mediterranean region," and related terms were used to ensure a thorough search. Inclusion criteria were defined to ensure the selection of studies aligning with the review objectives. These criteria included studies conducted in the Mediterranean region, focusing on cosmeceutical properties of medicinal plants, and published in English. Relevant data from the selected studies were extracted systematically, including information, such as the study objectives, plant species studied, extraction methods used, and cosmeceutical properties reported. Exclusion criteria were established to exclude studies that do not meet the specific requirements of the review, such as studies on non-Mediterranean plants or those unrelated to cosmeceutical applications, and the eligibility of the included studies was judged using the PRISMA checklist, Fig. 1. The botanical names of the species taxa were validated using the World Flora Online (WFO, www.worldfloraonline.org, accessed on February 2025) database.

## Mediterranean cosmetology: an updated perspective

Mediterranean cosmetology continues to evolve and adapt to new trends and technologies in the beauty industry. Recently, there has been a growing focus on natural and organic ingredients, such as olive oil and Henna, and traditional techniques, such as traditional baths (hammam), massages, and herbal remedies, to promote healthy and radiant skin and hair [19]. The Mediterranean cosmetology is a great example, with consumers being even more conscious of the impacts of synthetic beauty products on human health and environment. Indeed, Mediterranean cosmetology offers a sustainable and eco-friendly alternative that is rooted in the centuries-old wisdom of the Mediterranean cultures [20]. Some recent developments and trends in Mediterranean cosmetology can be emphasized into three main points:

- The use of natural and organic ingredients: There is a growing trend towards using natural and organic ingredients in Mediterranean cosmetology, in response to the consumers’ demand for cleaner and safer beauty products.
- Adoption of advanced technologies: While traditional practices continue leading the Mediterranean cosmetology, numerous brands are also incorporating cutting-edge technologies to enhance the effectiveness of their products and treatments.
- Emphasis on sun protection: Mediterranean countries are known for their sunny climates, which can cause skin damage. As a result, many Mediterranean beauty brands now offer sunscreens and other products that help protect the skin from UV rays [21].

## Traditional and modern aspects of plants and their derivatives as a source of cosmeceuticals in the Mediterranean Region

Mediterranean Region has a rich variety of plant life with a high biodiversity. Medicinal and aromatic plants have been traditionally used in local cuisine for seasonings as well as to relieve patient suffering and to cure diseases. The high diversity could be expounded by the exclusive climate and geography of the Mediterranean region resulting in the presence of both indigenous and endemic species, as well as those introduced through migration, trade, and occupation [22].

Diverse cosmeceutical applications of medicinal plants in the Mediterranean area have been listed, Table 1. Through the extraction and purification of bioactive natural compounds, these plants contribute to the development of novel cosmeceutical formulations.

**Table 1:** Cosmeceutical uses of medicinal plants in the Mediterranean area

Vernacular names	Scientific name	Used parts	Preparation	Key cosmeceutical uses	Country	References
Apiaceae						
*Addaryas*	*Smyrnium Olusatrum* L.	L	Powder	Wound healing	Morocco	[24]
*Accio*	*Apium graveolens* L.	R, S	Decoction	Dandruff, bruises, contusions, wounds	Turkey	[23]
*Pastenaca*	*Daucus carota* L. subsp. *Sativus*	R	Raw	Pimples, wounds, dermatitis		
*Fenucchiello*	*Foeniculum vulgare* Mill	R	Fumigation of decoction	Eye pains		
*Petrusino*	*Petroselinum crispum* (Mill.) Fuss	AP, L	Decoction	Bruises, burns, pimples, wounds		
Araliaceae						
*Ellera*	*Hedera helix* L.	L	Decoction	Acne, hair loss, bruises, burns, skin rashes, wounds, varicose veins	Turkey	[23]
Apocynaceae						
*Dafla*	*Nerium oleander* L.	L	Decoction^α^, Infusion^β^, poultice^β^	Skin diseases^α^,dandruff^β^, leprosy^β^	Algeria^**α**^,Morocco	[24, 25]
Asteraceae						
*Chiba*	*Artemisia absinthium* L.^a^	L	Infusion^α^, Powder mixed with oils^β^	Otitis^α^, skin infections^β^, anti-wrinkle^β^	Morocco	[26]
*Chih*	*Artemisia herba-alba* Asso	L	Poultice	Wound healing		
*Oum lbina*	*Launaea arborescens* (Batt.) Murb	S	Latex	Wounds and acne, skin care		
*Chbartu*	*Senecio anteuphorbium* (L.) Sch.Bip	S	Poultice	Wound healing		
*Qranfel*	*Syzygium aromaticum* (L.) Merr. & L.M.Perry	Clo	Powder^α^, poultice^β^, Decoction^γ^	Toothache^α^, haircare^β^, bad breath^γ^		
*Chih*	*Artemisia herba-alba* Asso	L	Poultice	Wound healing	Morocco	[27]
*Addad*	*Atractylis gummifera* Salzm. ex L	R	Decoction^α^, poultice^β^	Skin care^α^, hair care^β^		
*Tafsa*	*Bubonium graveolens* (Forssk.) Maire	S	Raw	Dental hygiene (brushing)		
*Oum lbina*	*Launaea arborescens* (Batt.) Murb	S	Latex	Wounds and acne, skin care		
*Chbartu*	*Senecio anteuphorbium* (L.) Sch. Bip	S	Poultice	Wound healing		
*Lmghayzli*	*Volutaria crupinoides* (Desf) Maire	L	Powder	Jaundice		
*L'jamra*	*Calendula officinalis* L.	L	Powder	Wounds, burns	Morocco	[28]
*Taymat*	*Cynara humilis* L.	R	Powder	Wounds, burns		
*Magraman*	*Dittrichia viscosa* (L.) Greuter	L	Powder	Wounds, burns		
*Khasse*	*Lactuca sativa* L.	L	Mixed with cucumber, turnip, and carrots	Mask for face care		
*Civanperçemi*	*Achillea millefolium* L.	AP	Decoction	Wound healing	Turkey	[29]
*Elbaboundj*	*Matricaria chamomilla* L.	L	Infusion	Skin diseases	Algeria	[30]
*Achiba*	*Artemisia arborescens* L.	L	Powder	Wrinkles and skin infections	Morocco	[31]
*Addad*	*Atractylis gummifera* L.	R	Powder	Skin abscesses and warts		
*Cardenzol*	*Centaurea ornata* Willd	R	Decoction	Furuncles	Portugal	[32]
*Erva-montã*	*Pulicaria odora* (L.) Reichenb		Cataplasm	Wounds		
*Erva-da-talasma*	*Senecio jacobaea* L.	L	Cataplasm	Furuncles		
Seca-ossos						
*Loloucha*	*Calendula arvensis* M.Bieb	L, Fr	Cataplasm, infusion	Burns, wound healing	Algeria	[25]
*Djamra*						
*Lappa*	*Arctium lappa* L.	L	Leaves juice mixed with the juice of *Urtica* spp. whole plant	Acne, dry skin, pimples, hair loss, varicose veins	Italy	[23]
*Servatica*	*Cichorium intybus* L.	AP, L	Decoction	Acne, wounds	Italy	[23]
*Cecoria*						
*Cardo*	*Silybum marianum* (L.) Gaertn	WP, R	Decoction	Wounds		
*Piscialietto*	*Taraxacum campylodes* G.E. Haglund	L	Infusion	Skin inflammation, varicose veins, warts, varicose veins		
*Cianfa*	*Tussilago farfara* L.	R, L	Decoction with *Malva sylvestris* leaves	Acne, dry skin, skin infections, skin infections, pimples, wounds		
*Tefaf*	*Sonchus oleraceus* L.	L	Decoction	Warts	Algeria	[25]
Brassicaceae						
*Çingirdak*	*Capsella bursa-pastoris* (L.) Medik	Capit	Infusion	Wounds	Turkey	[36]
*Şingirdak otu*						
*Tahtaci otu*						
*Eşekdikeni*						
*Vruoccolo*	*Brassica oleracea* L.	L	Crushed leaves mixed with olive oil	Pimples, contusions, burns, skin inflammation, shoulder pains, varicose veins	Italy	[23]
*Rafaniello*	*Raphanus raphanistrum* L.	R	Raw	Greasy skin		
*Al girjir*	*Eruca sativa* Miller	WP	Lotion	Wounds	Morocco	[24]
*Sibryan*	*Sisymbrium irio* L.	LS	Poultice	Wound healing		
Cactaceae						
*EL Hendi*	*Opuntia ficus-indica* (L.) Mill		Cataplasm	Skin diseases	Algeria	[25]
Caprifoliaceae						
*Fiocco ‘e cardinale*	*Centranthus ruber* (L.) DC	L	Infusion, decoction	Hair loss	Italy	[23]
Crassulaceae						
*Basilios*	*Umbilicus rupestris* (Salisb.) Dandy	L	Poultice^α^, infusion^β^	Burns^α^, acne^β^, warts^β^	Spain	[37]
*Vasillos*						
*Hoja de llaga*						
Cucurbitaceae						
*Cetrulo*	*Cucumis sativus* L.	Fr	Raw	Skin diseases, wrinkles, pimples, face redness	Italy	[23]
Ericaceae						
*Sovera pelosa*	*Arbutus unedo* L.	L, Fr	Decoction	Wounds	Italy	[23]
Fabaceae						
*Retam*	*Retama raetam* (Forssk.) Webb & Berthe	AP	Cataplasm	Wounds	Algeria	[25]
*Feniello*	*Trigonella foenum-graecum* L.	AP	Mixed with egg yolk	Dry skin, rashes, warts	Italy	[23]
Fagaceae						
*Cerza*	*Quercus pubescens* Willd	Bark	Decoction	Skin diseases, wounds, varicose veins	Italy	[23]
Geraniaceae						
*Nicchinocco*	*Pelargonium peltatum* (L.) L’Hér	L	Topic use	Wounds, burns, pimples, skin diseases	Italy	[23]
*Nicchinocco*	*Pelargonium zonale* (L.) L’Hér. ex Aiton	L	Topic use	Wounds, burns, bruises, pimples		
Globulariaceae						
*Zriga*	*Globularia alypum* L.	L	Crushed and mixed with milk as an ointment	Furunculosis	Tunisia	[38]
Lamiaceae						
*Chendgura*	*Ajuga iva* (L.) Schreb	L	Poultice^α^, decoction^β^	Hair care^α^, skin care (rinsing)^β^	Morocco	[27]
*Lokhzama beldiya*	*Lavandula dentata* L.	LS	Decoction	Bad breath		
*Halhal*	*Lavandula stoechas* L.	LS	Poultice	Hair loss		
*Lmrot*	*Marrubium vulgare* L.	L	Poultice	Wounds		
*Timija*	*Mentha suaveolens* Ehrh	L	Decoction	Toothache		
*Manrubio negro*	*Ballota nigra* L.	FAP	NS	Toothache	Spain	[33]
*Manrubio fétido*						
*Alhucema*	*Lavandula latifolia* Medicus	Fr, FAP	NS	Wounds, ulcers, contusion, animal bites, eczema	Spain	
*Espliego*						
*Alucemón*						
*Cantuezo*	*Lavandula stoechas* L.	FAP, Fr	Infusion	Wounds, ulcers, contusion, animal bites, eczema	Spain, Morocco	
*Tomillo*						
*Cantueso basto*						
*Habaq*	*Melissa officinalis* L.	AP	NS	Halitosis	Morocco Spain	
*Kezouân*						
*Badrendjouya*						
*Turunjân*						
*Narpuz*	*Mentha pulegium* L.	AP	Infusion, decoction	Sunburn	Turkey	[34]
*Yarpuz*						
*Biberiye*	*Rosmarinus officinalis* L.	AP	Infusion	Wounds, anti-aging, skin diseases		
*Almakeyik*	*Salvia fruticosa* Miller	L	Infusion	Skin diseases, burns		
*Almageyik*						
*karakekik*	*Satureja thymbra* L.	AP	Decoction	Gingivitis		
*Peynir keki ˘gi*						
*Kekik*						
*Taş nanesi*	*Micromeria fruticosa* (L.) Druce subsp.*barbata* (Boiss. Et Kotschy) Davis	AP	Decoction	Inflamed or suppurating wounds	Turkey	[35]
*Khzâma*	*Lavandula angustifolia* Mill	L	Powder	Wounds, hair loss	Morocco	[28]
*Merdedouche*	*Origanum majorana* L.	L	Powdered plant mixed with *Trigonella foenum-greacum*, *Peganum harmala*, and olive oil	Hair loss		
*Rtaimya*	*Sideritis incana* L.	AP	Bath	Skin allergy	Algeria	[25]
*Liliaceae*						
*Rusco*	*Ruscus aculeatus* L.	Rh	Decoction	Varicose veins	Spain	[37]
*Cornicabra*						
*Lythraceae*						
* Henna*	*Lawsonia inermis* Roxb	L	Mixed with water	Hair tonic	Morocco	[31]
*Malvaceae*						
*Malva*	*Malva sylvestris* L.	Fr, L	Cataplasm,	Wounds, furuncles	Portugal	[32]
* Ebe gömeç*	*Malva neglecta* Wallr	L	Crushed leaves mixed with milk	Wounds	Turkey	[36]
* Gömeç*						
* Bissam*	*Hibiscus sabdariffa* L.	Fr	Infusion	Hair care	Morocco	[27]
*Oleaceae*						
* Zeytin*	*Olea europaea* L.var.* europaea*	L	Mixed and cooked with olive oil	Wounds	Turkey	[36]
*Poaceae*	*Avena algeriensis* L.	Fr	Decoction	Skin whitening	Algeria	[39]
*Tiliaceae*	*Corchorus olitorius* L.	FAP	Raw	Hair loss	Algeria	[39]

*NS* not specified, *Clo* cloves, *S* stems, *LS* leafy stem, *FAP* flowered aerial parts, *F* flowers, *Fr* fruits, *WP* whole plant, *Rh* rhizome

^a^Herbarium number was not mentioned in the study

Among the various plants studied to date, those belonging to the family of *Asteraceae*, *Apiaceae*, *Brassicaceae*, *Lamiaceae*, and *Malvaceae* have been the most widely investigated, with leaves, aerial parts, and stems being the plant parts with the highest abundance of bioactive molecules. The countries from whom these Mediterranean plants have been more frequently collected for in-depth studies include Morocco, Turkey, Algeria, Italy, Spain, Portugal, and Tunisia. The referred plant parts are composed of a rich variety of biomolecules with renowned antioxidant, antimicrobial, UV protection, anti-aging, anti-pollution, moisturizing, smoothing, and anti-hyperpigmentation properties being thus of extreme usefulness for cosmeceutical purposes. Their wound healing potential, along with the ability to promote skin and hair care, namely acting on acne, hair loss, burns, skin rashes, wrinkles, pimples, varicose veins, furuncles, eczema, ulcers, sunburn, bruises, and also promoting dental hygiene make them a source of growing interest for study thanks to the multiple potentialities of the key molecules present in their chemical composition for upcoming applications, Table 1.

Medicinal plants belonging mainly to *Apiaceae* family: *Smyrnium Olusatrum* L., *Apium graveolens* L., *Daucus carota* L. subsp., *Sativus*, *Foeniculum vulgare* Mill and *Petroselinum crispum* (Mill.) Fuss are used in Morocco and Turkey to heal wounds bruises, contusions, pimples, dermatitis, relieve eye pains and eliminate dandruff. [23, 24] Likewise, in Turkey, women use *Hedera helix* L. leaves to prevent hair loss, bruises, alleviate burns, skin rashes, wounds and varicose veins. [23–25] It is mentioned that in Morocco and Algeria women use *Nerium oleander* L. leaves to cure skin diseases and to treat dandruffs and leprosy. *Asteraceae* is the largest plant family used mostly in Morocco, Turkey, Portugal, Italy and Algeria to remedy skin infections, anti-wrinkle, wounds healing, toothache, hair care, bad breath, burns, acne, furuncles, dry skin, and pimples. [23–32] *Brassica oleracea* L from *Brassicaceae* used in Italy to remedy contusions, burns, skin inflammation, shoulder pains, varicose veins. [24] *Lamiaceae* has been used in Mediterranean area largely in Morocco, Spain and Turkey for hair care, skin care, toothaches, wounds, ulcers, contusions, animal bites, eczema, halitosis, sunburn, anti-aging, burns. [27, 28, 33–35] Other medicinal plants belonging to different families which have been used as traditional cosmetics for hair and skin care are listed in Table 1.

## Current evidence on the use of Mediterranean plants for cosmetic drives

Medicinal plants contain various chemical compounds that have pharmacological properties, meaning they can interact with the human body and potentially provide therapeutic effects. Quite a lot of researches underlining that many Mediterranean plants hold excellent pharmacological properties making them key candidates for cosmetic applications [40].

Understanding the cosmetic plants potential effect can help in the development of effective and safe cosmetic products. In Table 1, the most popular medicinal plants used for cosmetic proposes in the Mediterranean region are enumerated. Their pharmacological properties were verified. We notice that most of the properties that have been confirmed for these medicinal and aromatic plants are similar, which can be explained by their chemical composition, as they contain mostly the same secondary metabolites.

Taking into consideration the information presented above, it should be noted that most studies currently carried out had the main triggers on the popular knowledge related to their applications for multiple purposes. In fact, and despite conceiving this knowledge as empirical, from the studies carried out to date, as shown in Tables 1 and 2, it can be emphasized that most pharmacological properties attributed to these plants as a result of the empirical knowledge are now confirmed. However, until the establishment of the research in the area, its mode of action and the main metabolites responsible for a certain effect were unknown.

**Table 2:** Pharmacological evidences of cosmetic plants in Mediterranean area

Scientific name	Family	Part of plant	Plant properties	References
*Olea europaea* L.	Oleaceae	Leaf	Antimicrobial; antioxidant	[106–109]
*Aloe vera*(L.) Burm. f	Aloeaceae	Leaves (Aloe vera gel)	Antimicrobial; antifungal; Antioxidant; anti-inflammatory	[110, 111]
*Calendula officinalis* L.	Asteraceae	Flowers	Antioxidant; anti-inflammatory; antimicrobial	[112–114]
*Thymus vulgaris* L.	Lamiaceae	Leaves	Antioxidant; anti-inflammatory; antimicrobial	[115–117]
*Rosmarinusofficinalis* L.	Lamiaceae	Leaves	Antimicrobial; antioxidant; anti-inflammatory	[118–120]
*Trigonellafoenum-graecum* L.	Fabaceae	Leaves	Antimicrobial; antioxidant; anti-inflammatory	[121–123]
*Lawsoniainermis* Linn	Lythraceae	Leaves	Antimicrobial; antioxidant; anti-inflammatory	[124, 125]
*Rosa x damascena* Mill	Rosaceae	Flower Extract	Antioxidant; anti-inflammatory	[126, 127]

Today, it is widely accepted that the various secondary metabolites present in its composition are the main responsible for the pharmacological effect, and that, contrary to what was thought, the final bioactive effect does not result only from the effect of the most abundant compounds, but rather from the interaction of those with vestigial compounds. This finding was one of the main drivers able to understand why many drugs do not act effectively when used alone, even at high doses, but are highly effective at low ones when combined with other active principles [41].

Nowadays, interest in the employment of plant extracts is becoming more and more important because of their richness in bioactive compounds, such as polyphenols and carotenoids, to whom extremely important bioactive effects have been listed. [42, 43] Phytochemicals are widely abundant in plants and play important roles, not only for maintain the good health of the plant, but also to protect it from invaders, and secondarily, provide to humans extraordinary phytopharmacological effects, thus acting on prevention, monitoring and even treatment of a certain ailment [44].

Plants with medicinal properties and health benefits are numerous. Amongst the secondary metabolites elaborated by the plants from the Mediterranean region to protect themselves against invaders, terpenoids, alkaloids, sulphur-containing compounds and phenolic compounds comprise the most widely studied and representative, owing to their multiple biological effects. The latter comprise a large group of naturally-occurring bioactives with excellent pharmacological properties, such as antimicrobial, anti-carcinogenic, antioxidant, anti-inflammatory, antiviral, antitumor, anti-aging, and neuroprotective properties [45–47]. Alkaloids and terpenes are also present, despite less abundantly, and have remarkable antioxidant, antibacterial, and anti-inflammatory activities [48]. Although their beneficial properties are remarkable, the overuses of phytochemicals have to be considered because of their toxicological attributes. In fact, in traditional medicine, the amount and frequency of using the phytochemicals is frequently not controlled and the risk of excessive of consumption with potentially harmful effects is eminent [49]. Thus, proper regulations and elucidation approaches that natural does not mean safe should be adopted.

In the following subsections, a brief overview on the pharmacological effects of customarily used plants from the Mediterranean region for cosmetic purposes is given.

### Olive oil (*Olea europaea *L. var. *europaea*)

*Olea europaea* belongs to the Oleaceae family and is commonly known as fruit oil or just olive oil and is the hale and hearty oil used by millinery. In fact, the ancient Mediterranean civilizations were using this natural essential oil, among others, in food and for dermatologic applications, thermal massage, hair care, and for healing wounds [50, 51]. In Mediterranean countries, olive oil is widely applied as photoprotective agent to protect skin and keeping it young and healthy due to its richness of antioxidant compounds [52]. Among other important proprieties, the anti-inflammatory and antioxidant activity displayed by olive oil advocated its effectiveness for dermatologic applications. In fact, several cosmetic products recently contain olive oil as one of its main ingredients, like shampoos, hair conditioners, skin care products, bath soaps, makeups, etc.

### Argan oil (*Argania spinosa* L.)

Argan oil is exclusively grown and produced commonly in Southwestern Morocco [53]. It is a popular ingredient in hair care products used to moisturize and nourish the hair, also used to eliminate skin pimple and reduce dryness and prevent the appearance of skin wrinkles [54, 55]. Because of its richness in vitamin E, unsaturated fatty acids, phytosterols, tocopherols, and volatile compounds, the cosmetic cold pressed argan oil is applied straightly on the body or added as a main constituent in cosmetic formulations [56]. In addition, the fatty-acid profiling could be useful as a determining factor of quality and origin of cosmetic argan oil. In fact, a study showed that oleic acid (C18:1), linoleic acid (C18:2), and palmitic acid (C16:0) are respectively the main fatty acid in cosmetic argan oil [57].

### *Aloe vera* (*Aloe barbadensis* Miller)

*Aloe vera* is a highly valued medicinal herb owing to its extensive history of use in skincare and beauty practices [58]. It is a reserve of bioactive compounds, including minerals, amino acids, polysaccharides, vitamins, and sterols [59, 60]. These compounds have demonstrated remarkable effects on the skin, such as moisturizing and preventing skin dryness, reducing inflammation and redness, promotion wound healing, protection against free radical-induced skin damage, stimulation of hair growth, and treatment of dandruff. The appliance of *A. vera* in skincare is deeply rooted in traditional practices, with ancient Egyptians relying on it to treat burns and wounds, and ancient Greeks harnessing its potential for promoting hair growth. Recent scientific studies have further substantiated the potential benefits of *A. vera* for skin health, with evidence supporting its efficacy in addressing various skin conditions, including acne, eczema, psoriasis, sunburn, and wounds [59, 61]. Its skin-enhancing properties can be attributed to its polysaccharide content, such as Acemannan (a D-isomer mucopolysaccharide), which exhibits notable moisturizing and protective assets [62]. The synthesis of hyaluronic acid, a natural humectant responsible for maintaining optimal skin hydration, is boosted by the polysaccharides present in the plant [62, 63]. Hyaluronic acid plays a crucial role in preserving moisture and promoting skin plumpness. However, natural aging processes can lead to a decline in endogenous hyaluronic acid production, resulting in cutaneous dryness and wrinkle formation. *A. vera* has emerged as a potent botanical agent capable of stimulating the production of hyaluronic acid, thereby enhancing skin hydration, and fostering a more youthful appearance [59]. *A. vera* can stimulate the elastin and collagen synthesis, crucial proteins involved in maintaining skin elasticity and structural integrity, while decreasing the growth of various pathogens and then dipping the risk of wound infections. [64, 65] In addition to its polysaccharide content, *A. vera* possesses other bioactive components that contribute to its beneficial effects on the skin. Among other phytoconstituents of *A. vera*, vitamin C has been displayed to exert a melanin-reducing effect through inhibiting tyrosinase, tyrosine hydroxylase, and dopa oxidase, hence helping to regulate skin pigmentation [66, 67]. As for its mineral composition, including zinc and magnesium, it performs in reducing inflammation and promoting wound healing. Zinc, specifically, acts by inhibiting the inflammatory cytokines production [68]. Additionally, the amino acids present in *A. vera* contribute to hair growth promotion and dandruff treatment. These amino acids work on as the elementary units for proteins, stimulating the production of keratin, an essential protein for hair strength and structure [69]. The diverse and multifaceted assets of *A. vera* highlight its potential as a promising ingredient in skincare and hair care formulations.

### Calendula (*Calendula officinalis* L.)

*Calendula officinalis* L., or pot marigold, from Asteraceae family, is a flowering plant that is endemic to Europe and Asia characterized by its cheerful orange or yellow flowers. Calendula has been used for centuries for its medicinal properties, and it is now widely used in cosmeceutical products [70]. It is traditionally used as sleep inducing, to promote menstruation and to increase perspiration. Up-to-date, its leaves are applied on lesions and the capitulum extract is good to heal burns [71]. This plant has emerged as a prominent cosmeceutical ingredient, combining both cosmetic and pharmaceutical properties, thus fueling its recent surge in popularity. This is attributable to the presence of multiple phytobioactive compounds, especially flavonoids, polysaccharides, amino-acids, carotenoids, and triterpenoids, among others, which confer calendula with remarkable skincare benefits [72]. Flavonoids in calendula play a pivotal role as potent antioxidants, effectively shielding the skin from free radicals-induced harm and inhibiting the production of melanin involved in hyperpigmentation [73]. A present-day study examined the ethyl acetate extract of the pot-marigold flower petals influence on melanoma and revealed that this extract demonstrated significant anti-melanogenic and anti-migration activities in melanoma cells. Thus, it offers a promising avenue for the skin cares [74]. Another investigation suggested that the oral doses of *C. officinalis* hydroalcoholic extract at 150 and 300 mg/kg effectively prevented UVB irradiation-induced depletion of glutathione (GSH), a vital enzyme in restoring cellular redox homeostasis and protecting against oxidative injury [75]. This protective effect against UVB-induced photo-aging and skin harm was ascribed to the presence of flavonoids, especially rutin and narcissin, which denotes the potential of *C. officinalis* extract as a valuable skin prevention strategy against the damaging effects of UVB radiation [76].

### Thymus (*Thymus vulgaris* L.)

*Thymus vulgaris* L. is an ornamental and edible plant from the mint family *Lamiaceae* cultivated mainly in the Mediterranean region. This medicinal herb is mainly rich in monoterpenes, *p*-cymene, carvacrol, and thymol, and has been used since ancient times, for example, as disinfectant, anthelmintic, antibiotic, carminative, antifungal, antiseptic, refresher [77–81]. Owing to its antioxidant activities, the essential oil was largely used to alleviate rheumatoid-arthritis and stiff joints pains [80]. As well, it has been used to remedy dermatosis, like acne, eruption, seborrhea and for relieving skin irritations [82].

### Rosemar*y* (*Rosmarinus officinalis* L.)

This aromatic plant from the *Lamiaceae* family is among the finest medicinal plants used in Mediterranean region for medicinal, culinary, and cosmetic applications due to its richness on antioxidant phytochemicals and secondary metabolites [83]. From the ancient world of Mediterranean region, the essential oil and distillated water of rosemary were employed to prevent some dermatitis, to protect skin against high temperatures, as pain-relieving, as eyewash to treat common eye infections, and as general refresher [84–86].

### Fenugrec (*Trigonella foenum-graecum* L.)

Fenugreek is an endemic plant belonging to the Fabaceae family. It is growing in the Mediterranean area and mainly used to treat insufficient breast milk for postnatal mothers and to increase weight gain for new natal [87]. Fenugreek is appreciated for its medicinal and dietary properties in line with the richness of the seeds on phytochemicals. [88, 89] Widely recognized for its excellent therapeutic abilities against diabetes and hypercholesterolemia, digestive disorders, studies reported that Fenugreek seeds possess several proprieties like antioxidant, antimicrobial, anti-inflammatory, and antitumor, among others, due the presence of vanillic, coumaric, ferulic, and gallic acids, known for their important antioxidant activities [90–92]. Additionally, the extracts of the seeds were used in the north of Africa to reduce hair loss [93]. Latest research displayed that the ethanolic Fenugreek extract can reduce skin aging. In fact, it has displayed that the nanoencapsulated ethanolic Fenugreek extract can improve portentously the molecular mechanisms against skin-aging putting forward the potential use of Liponiosome encapsulating fenugreek extract for transdermal delivery [94].

### *Henna* (*Lawsonia inermis* Linn.)

Henna (*Lawsonia inermis* Linn.) is a part of the family Lythraceae renowned for centuries in the Ayurveda for cosmetic applications, especially owing to its useful properties on skin and hair. Badoni Semwal et al., mentioned that Henna has different medicinal uses. In fact, the extract exhibits wound healing, hypoglycemic, antioxidant, hepatoprotective, antibacterial, antifungal, antiviral, antimycotic effects [95]. In Mediterranean region, the leaves rich on hennotannic acid, (2-hydroxy-1,4-naphthoquinone) are mainly used to produce a natural dye for hair, skin, and nails and to create intricate temporary tattoos to adorn hands and feet [96].

### *Damask rose *(*Rosa *×* damascena Mill*.)

Damask rose known also as Damascus rose or Rose of Castile is an ornamental plant belonging to the Rosaceae family with perfuming effect. It is a hybrid rose (*Rosa gallica* × *Rosa phoenicia*) raised in the Mediterranean countries and traditionally used to produce rose water besides its widespread application for perfuming hair and repair skin [97, 98]. It possesses a number of therapeutic applications, such as antimicrobial, antiseptic, anti-inflammatory, antidepressant, analgesic, antipyretic, and stomachic effects, besides acting as a general tonic. Its flowers are used to treat itching on skin, throat infections, and pains. *Rosa x damascena* is commonly used to treat skin problems and protect it against UV radiation [99]. In addition, the flowers are used in combination with herbals for preservation of aging by boosting the collagen production and improving the skin glowing [100, 101].

### *Artemisia absinthium* L.

Commonly known as wormwood, *Artemisia absinthium* is an enduring aromatic plant employed for its medicinal properties for centurial years. In recent years, it has also been investigated for its potential cosmeceutical benefits. Plant-derived bioactive compounds have been shown to improve the skin quality, including reducing hyperpigmentation, improving skin elasticity, reducing the appearance of cellulite, shrinking large pores, treating acne, and preventing wound infections [102, 103]. According to the European database gathering data on cosmetic ingredients, Cosmetic Ingredient database agrees the use of *A. absinthium* in cosmetics products like skin care products, namely cleanser moisturizes, serums, refreshers, and eye mask, and hair care products, such as shampoos, conditioners and styling, as natural antiseptics and for fragrances [104, 105].

## Clinical evidence of Mediterranean plants for cosmetic drives

Medicinal plants used in cosmetics in Mediterranean Region present good anti-oxidant and anti-inflammatory activities, Table 2. Clinical trials were performed to evaluate the plant extract activities and to prove the traditional uses of those plants, Table 3. In fact, the treatment of the skin with a hydrogel containing *Olea europaea* L. leaf extract for 8 weeks with an application of three days per week balances and promotes the wound healing, modulate reactive oxygen species and pH, and pain relief properties. Its effectiveness is due to its multi-dose format application and biocompatibility [128]. Another study was performed to assess the usefulness of *Aloe vera* gel against radiation-induced dermatitis [129]. In fact, a double-blind trial using *Aloe vera* gel (98% pure) twice a day starting within three days of radiation initiation (ideally on the first day), revealed its effectiveness against radiation-induced dermatitis.

**Table 3:** Clinical evidence of medicinal plants and their derivatives for cosmetic purposes in Mediterranean area

Plant	Parts	Extract	Doses	Administration	Main effects	References
*Olea europaea* L.	Leaf	–	Treatment duration was set at 8 weeks unless complete healing was achieved previously. Product applications were to be carried out 3 days a week (24 applications in total at most) and, whenever possible, on alternate days	Skin Gel	Balances and promotes the healing of ulcer microenvironment Modulate reactive oxygen species and pH Pain relief properties Efficacy due to its multi-dose format	[128]
*Aloe vera* (L.) Burm. f	Leaves	Gel	Treatment field twice a day starting within 3 days of radiation initiation (ideally on the first day)	Aloe Vera Gel 98% pure	Usefulness of aloe-vera gel for prophylactic treatment of radiation-induced dermatitis High degree of correlation between patients and their health care providers regarding the grading of dermatitis	[129]
*Calendula officinalis* L.	Plant	Methanol extract	The patients apply the treatment once per day for 02 weeks	Herbal Cream	Formulation containing the mixed extract of Calendula officinalis, Rosa canina, Zataria multiflora, Trigonella foenum graecum and Glycine max Further assays to evaluate possible undesired effects of the formulation	[130–132]
*Thymus vulgaris* L.	Flower/Leaf	–	The volunteers applied on the facial skin twice a day, for 60 days	ThymLec Gel (Skin) (1.0–3.0%)	Anti-aging effects of a phytocosmetic preparation containing Thymus vulgaris associated with lecithin (ThymLec) ThymLec promoted a reduction of 10.2% in length and 7.0% in the area of perioral wrinkles at day	[133]
*Rosmarinus officinalis* L.	Leaves	Methanol extract	This experiment lasted 2 months by applying shampoo 3 times a week	Basic shampoo containing methanolic extract	Combination of medicinal plant extracts with Pirocton Olamine and Zinc-PCA in the shampoo form to treat dandruff on hair Remarkable dan-druff decrease and itching in the first week. Dandruff was highly removed	[134]

Phase II clinical trials were conducted using formulations of extracts containing *Aloe barbadensis*, Miller, *Azardirachta indica*, Juss, *Curcuma longa*, Linn, *Hemidesmus indicus*, Linn, *Terminaliachebula*, Retzr, *Terminalia arjuna*, Rob, and *Withania somnifera*, Linn mixed in different portions to treat acne. The treatment was applied twice daily during four weeks. According to the study, the integration of internal and external formulation preferably using topical cream formulation containing active ingredients than gel demonstrated its efficiency compared to the use of internal preparation solely. Other studies underlined the efficacy of a formulation containing mixture of medicinal plants to treat acne vulgaris and acne-induced inflammation lesions sidestepping microbial resistance to chemical medicines [130–132]. ThymLec Gel continent (1.0%-3.0%) of *Thymus vulgaris* L. flower/leaf applied on the facial skin twice a day for 60 days promoted a reduction of 10.2% in length and 7.0% in the area of perioral wrinkles at day [133]. Basic shampoo containing methanolic extract of *Rosmarinus officinalis L.* leaves used 3 times a week for 2 months decrease dandruff in the first week and highly removed it in the last week [134].

## Formulations used in cosmetic industry: pros, cons, and opportunities

Formulations used in the cosmetic industry refer to the various ingredients and substances used to elaborate cosmetic products, such as creams, ointments, serums, makeups, etc. While a critical component of the cosmetic industry, the various bioactive principles used for formulating cosmeceutical products have also several features that regulate the way the formulation can be done. In addition, each bioactive present its own characteristics of stability and bioavailability, which markedly determines the various ingredients that can be added while preparing the formulation. Thus, despite they offer multiple benefits, they also have their challenges. In this sense, it is imperative that the cosmetic industry establishes a balance between innovation, safety, and sustainability to meet the changing needs of consumers. Therefore, several advantages, drawbacks, and opportunities may be enumerated while formulating new products for cosmetic purposes, Table 4.

**Table 4:** Key factors in cosmetic industry

Pros	Cons	Opportunities
Improved efficacy*:* formulations can improve the efficacy of cosmetic products by ensuring that the right combination of ingredients is used to deliver the desired result. For instance, a moisturizing lotion with the right formulation can help keep the skin hydrated and supple	Safety concerns: some ingredients used in cosmetic formulations can be harmful to human health	Natural and organic formulations: there is a growing demand for natural and organic cosmetic formulations that use plant-based ingredients and are free of harmful chemicals
Customization: formulations allow cosmetic manufacturers to customize their products to meet the specific needs of different customers. For instance, a formulation for dry skin would include different ingredients than one for oily skin	Cost: developing and testing new formulations can be expensive, which can lead to higher costs for the end consumer	Sustainable formulations: cosmetic manufacturers are increasingly focusing on developing sustainable formulations that minimize the environmental impact of their products
Innovation: formulations drive innovation in the cosmetic industry, leading to the creation of new and unique products that cater to specific needs	Environmental impact*:* some formulations may have a negative impact on the environment due to the use of non-renewable or harmful ingredients	Personalized formulations: advances in technology and data analytics have made it possible to create personalized formulations that cater to the unique needs of each customer

If by one hand, the advances stated in the various techniques of formulation markedly contribute to a fast raise in stability, bioefficacy, and bioavailability of the active principles, ultimately contributing to the delivery of increasingly innovative and effective products with proper customization features, on the other hand, the safety concerns, environmental impact, and related cost of acquisition are considered the main drawbacks, that at the same time also limit the easy access to such products by the population. Contrary to the folk practices, nowadays-natural cosmetics are generally more expensive than the organic ones, thus limiting the access to individuals with low-middle sociodemographic index. Nonetheless, and in a way to overcome the main limitations stated to date, multiple intents have been done to formulate natural formulations that promote the sustainability of the planet and that at the same time are patient-specific, i.e., personalized according to the specific needs of a certain individual or group of individuals. Taking all these aspects in mind, this review, while highlighting the main advances stated in the area of Mediterranean cosmetology, also underline the main key points that deserve a particular attention at short-term in order to provide increasingly safer, effective, and less deleterious action both to the environment and consumers that aim to promote, maintain, and even improve beauty and general wellbeing.

## Potential wound healing properties of Mediterranean medicinal plants

The skin is the body's first line of defense, serving as a dynamic barrier that shields internal structures from external environmental stressors. However, exposure to factors such as ultraviolet (UV) radiation, visible light, and environmental pollutants can elevate the production of reactive oxygen species (ROS) [134, 135]. This oxidative imbalance can compromise skin integrity, accelerate aging, and hinder the natural wound-healing process. Wound healing is a complex biological process essential for restoring skin integrity after injury. It involves a series of coordinated cellular and molecular events, including hemostasis, inflammation, proliferation, and remodeling [136, 137]. Delayed or impaired wound healing can lead to severe complications, such as infections and chronic wounds, increasing healthcare burdens worldwide [138].

On the other hand, medicinal plants have been extensively explored for their wound-healing properties, offering a natural and effective alternative to conventional treatments [139]. Their bioactive compounds, including flavonoids, alkaloids, tannins, terpenoids, and saponins, exhibit antimicrobial, anti-inflammatory, antioxidant, and tissue-regenerative activities, making them valuable in wound management [139]. The wound healing process requires a delicate balance between pro-inflammatory and anti-inflammatory responses, cellular proliferation, and extracellular matrix (ECM) remodeling. Several medicinal plants facilitate these processes through distinct mechanisms, including antimicrobial, anti-inflammatory, collagen synthesis and tissue regeneration.

### Antimicrobial activity

The skin's microbiome plays a vital role in defending against pathogens, but disruptions and pathogenic invasions can lead to infections, which not only hinder wound healing but also contribute to antibiotic resistance [140]. Medicinal plants possess potent antimicrobial properties that target a broad spectrum of wound pathogens, predominately *Staphylococcus aureus* and *Pseudomonas aeruginosa*. Their bioactive compounds, including tannins, flavonoids, and essential oils disrupt bacterial membranes, inhibit quorum sensing, and prevent biofilm formation, reducing the risk of infection and supporting faster wound closure [141]. In a recent study, the antibiofilm potential of essential oils from Tunisian *Ammi visnaga* L. fruits and *Foeniculum vulgare* Mill. aerial parts were tested against various Gram-negative pathogenic strains involved in chronic wound remodeling, including *P. aeruginosa* (DSM 50071), *Escherichia coli* (DSM 8579), and *S. aureus* subsp. *aureus* Rosenbach (ATCC 25923), using the crystal violet method. The MIC values ranged from 5 ± 2 to 10 ± 2 μl/mL, except for *S. aureus* (MIC > 20 μl/mL). The essential oils exhibited significant biofilm inhibition, reducing biofilm formation by up to 53.56% in *E. coli*. Additionally, they demonstrated strong antibacterial activity by disrupting bacterial metabolism in *E. coli*. This antibiofilm potential was attributed to the inhibitory effects of butanoic acid, 2-methyl-, 3-methylbutyl ester, linalyl propionate, and *trans*-anethole on tyrosyl-tRNA synthetase [142].

**Fig. 2:**
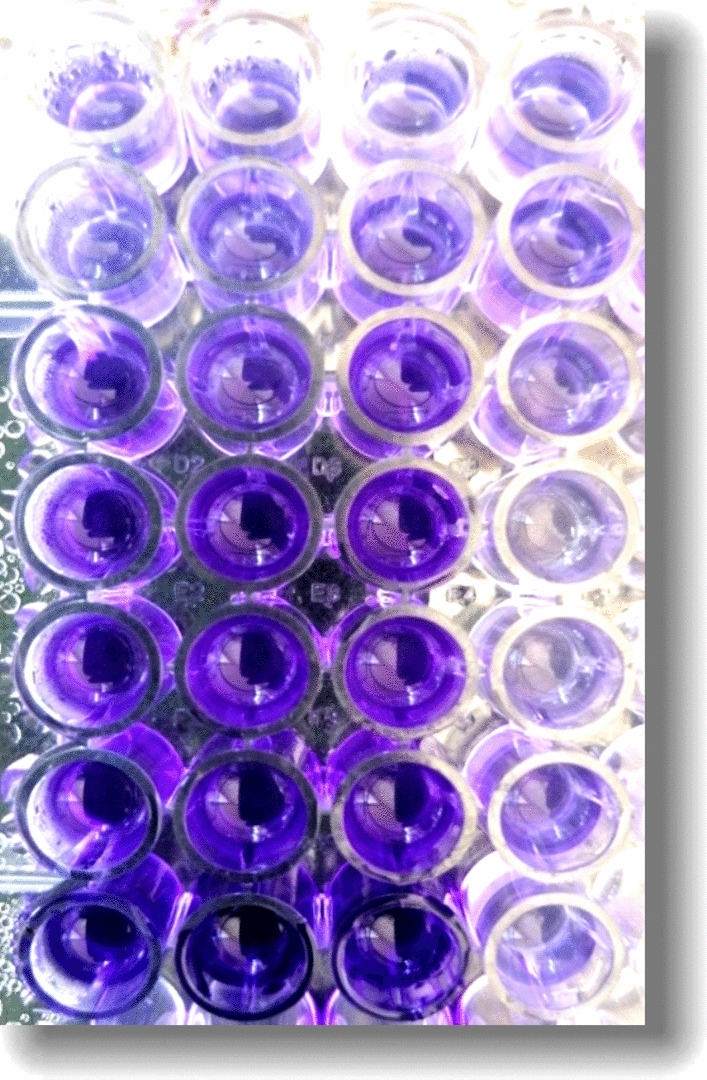
Antibiofilm potential of the essential oil from the aerial parts of Moroccan *Tetraclinis articulata* (vahl) mast ©2025

Furthermore, bacterial motility is a crucial virulence factor that enables pathogens to spread across surfaces, form biofilms, and colonize new environments, exacerbating infections. By facilitating movement over the skin and deep into wounds, motility enhances bacterial survival and resistance to treatment. Understanding the different types of bacterial movement, such as swimming, swarming, twitching, gliding, and sliding can help identify potential targets for developing more effective antibacterial therapies [143]. For instance, various medicinal plants from the Mediterranean region, including *Tetraclinis articulata* (Vahl) Mast. (Fig. 2) and *Piper cubeba*, effectively reduced the swimming and swarming motility zones of *P. aeruginosa* [144, 145].

### Skin photoprotection

Prolonged UV exposure accelerates skin aging, damages DNA, and increases the risk of skin cancer. Plant-derived metabolites, such as carotenoids and flavonoids help mitigate these effects by absorbing UV radiation and reducing its penetration into the skin [146]. Lycopene, found abundantly in tomatoes, is particularly effective in neutralizing singlet oxygen and dissipating excess energy as heat, preventing cellular damage. Beyond their photoprotective role, carotenoids such as *β*-carotene also serve as provitamin A, supporting skin cell proliferation and differentiation through the RAR/RXR pathway, which is crucial for wound healing. Carotenoid distribution in human skin varies, with the highest concentrations found on the forehead and palms. Factors like diet, UV exposure, smoking, and alcohol consumption influence skin carotenoid levels, with lycopene being particularly sensitive to UV-induced depletion [147].

### Anti-inflammatory activity

A controlled inflammatory response is critical for effective wound healing, as excessive or prolonged inflammation can hinder recovery. Bioactive compounds such as curcumin from *Curcuma longa* L. have demonstrated strong anti-inflammatory effects by downregulating pro-inflammatory cytokines, including tumor necrosis factor-alpha (TNF-α), interleukin-6 (IL-6), and cyclooxygenase-2 (COX-2). By regulating these pathways, medicinal plants help reduce oxidative damage, prevent tissue breakdown, and accelerate tissue repair [148].

## Challenges and perspectives of natural products in skincare and cosmetics

Despite the growing demand for natural ingredients in skin care and cosmetics, several challenges continue to hinder their widespread adoption and clinical use. In contrast to the rigorous testing and approval processes applied to pharmaceuticals, plant-based cosmetic formulations are not subject to the same level of regulatory scrutiny. The definition of terms such as "natural" and "organic" varies across different regulatory frameworks worldwide, resulting in inconsistencies in product labeling and quality control. Establishing universally accepted guidelines for natural skin care ingredients is critical to guarantee both consumer safety and product efficacy [149, 150].

Another major obstacle is the stability and bioavailability of plant-derived compounds. A considerable number of bioactive ingredients, including polyphenols and flavonoids, are susceptible to degradation when exposed to light, oxygen, or heat. This affects their efficacy and shelf life in cosmetic formulations. Advanced encapsulation techniques, such as liposomes and nanoparticles, are being explored to improve the stability and controlled release of these compounds, but these technologies remain costly and require further refinement for large-scale production [150, 151].

The efficacy of plant-based skincare products is another key consideration. While numerous natural compounds exhibit promising antioxidant, anti-inflammatory, and antimicrobial properties in vitro, their effectiveness in real-world applications depends on factors such as formulation, absorption, and interaction with other cosmetic ingredients. Rigorous clinical studies and in vivo research are needed to validate the dermatological benefits of medicinal plant extracts and optimize their incorporation into cosmetic products [152].

## Conclusion and viewpoints

In the Mediterranean region, the use of medicinal and aromatic plants in the daily routine is a common practice since antiquity. These plants are widely available, and have been used for centuries due to their numerous benefits for the skin and hair. Scientific research has identified some of the active compounds associated to the pharmaceutical properties of these traditional plants. Furthermore, clinical trials have shown that plant extracts can improve skin hydration, elasticity, and brightness, making them a promising alternative to synthetic cosmetic ingredients. Further research must be warranted to standardize plant extraction methods, assess their safety, efficacy, and determine their long-term effects.

Moreover, considering that the medicinal and aromatic plants usage represents a sustainable and natural approach for cosmetic industry, in line with the growing demand for eco-friendly and ethically-sourced products, increasingly deepen studies are vital to fully harness the benefits of these plants and identify their toxic effects, allowing a sustainable and effective application in cosmetic formulations.

Medicinal plants offer a multifaceted approach to wound healing by modulating inflammation, combating infections, neutralizing oxidative stress, and promoting tissue regeneration. As concerns over antibiotic resistance and limitations of synthetic wound-healing agents continue to rise, plant-based treatments provide a promising, natural alternative. In light of the mounting challenge of multidrug-resistant (MDR) infections, the development of alternative antimicrobial strategies is crucial. Future research should focus on optimizing the formulation and delivery of essential oil-based nanofibers to enhance their stability, bioavailability, and targeted antimicrobial action. Additionally, investigating their mechanism of action at the molecular level could provide insights into their efficacy against resistant pathogens. Comparative clinical studies with conventional treatments are also necessary to validate their therapeutic potential in human applications. Furthermore, exploring the synergistic effects of essential oils with existing antibiotics may offer promising avenues for overcoming bacterial resistance.

## Data Availability

No datasets were generated or analysed during the current study.
